# Antithymocyte globulin improves the survival of patients with myelodysplastic syndrome undergoing HLA-matched unrelated donor and haplo-identical donor transplants

**DOI:** 10.1038/srep43488

**Published:** 2017-03-06

**Authors:** Hong Wang, Hong Liu, Jin-Yi Zhou, Tong-Tong Zhang, Song Jin, Xiang Zhang, Su-Ning Chen, Wei-Yang Li, Yang Xu, Miao Miao, De-Pei Wu

**Affiliations:** 1 Jiangsu Institute of Haematology, The First Affiliated Hospital of Soochow University; Institute of Blood and Marrow Transplantation; Collaborative Innovation of Haematology; Key Laboratory of Thrombosis and Hemostasis of Ministry of Health, Suzhou, 215000, China

## Abstract

Significant advances have been achieved in the outcomes of patients with myelodysplastic syndromes (MDS) after both HLA-matched sibling donor transplants (MSDT) and non-MSDT, the latter including HLA-matched unrelated donor (MUDT) and haplo-identical donor transplants (HIDT). In this retrospective study, we analyzed the data of 85 consecutive patients with MDS who received allogeneic HSCT between Dec 2007 and Apr 2014 in our center. These patients comprised 38 (44.7%) who received MSDT, 29 (34.1%) MUDT, and 18 (21.2%) HIDT. The median overall survival (OS) was 60.2 months, the probabilities of OS being 63%, 57%, and 48%, at the first, second, and fifth year, respectively. Median OS post-transplant (OSPT) was 57.2 months, the probabilities of OSPT being 58%, 55%, and 48% at the first, second, and fifth year, respectively. The survival of patients receiving non-MSDT was superior to that of MSDT, median OSPT being 84.0 months and 23.6 months, respectively (P = 0.042); the findings for OS were similar (P = 0.028). We also found that using ATG in conditioning regimens significantly improved survival after non-MSDT, with better OS and OSPT (P = 0.016 and P = 0.025). These data suggest that using ATG in conditioning regimens may improve the survival of MDS patients after non-MSDT.

Despite the improvement in prognosis with the introduction of hypomethylating agents (HMAs)^1^,^2^, allogeneic haematopoietic stem cell transplantation (allo-HSCT) is still the only potentially curative treatment for selected patients with lower risk MDS and most patients with higher risk MDS. In general, HLA-matched sibling donors transplants (MSDT), which account for about 30% of transplants^3^, are the first choice in patients who are eligible for transplantation^4^,^5^. With the routine use of high-resolution HLA typing techniques, HLA-matched or mismatched unrelated donors have emerged as alternative donor sources for most patients without suitable HLA-matched sibling donors. Studies comparing MSDT and matched unrelated donors transplants (MUDT) have yielded conflicting results, some reporting inferior survival or disease-free survival (DFS) with MUDT^6^,^7^ and others reporting similar survival^8^,^9^. In recent years, haplo-identical donor transplants (HIDT) have been proved to be effective in patients with haematological malignancies, achieving comparable survival rates to those following MSDT or MUDT^10^,^11^.

However, one of the major drawbacks to non-MSDT (including MUDT and HIDT) is graft versus host disease (GVHD), which is the most important cause of death after allo-HSCT. Many approaches to minimizing GVHD complications after non-MSDT have been evaluated. Administration of antithymocyte globulin (ATG) during the conditioning regimen to promote *in vivo* T cell depletion of the graft is of particular interest^12^,^13^,^14^. Several retrospective studies have reported clinical outcomes of HIDT with an ATG-based regimen that are comparable to those of MSDT and MUDT^15^,^16^. Researchers from Zhejiang University, China prospectively compared the clinical outcome of 305 patients with haematological malignancies who underwent MSDT, MUDT, and HIDT and found that the incidence of GVHD and transplant-related mortality (TRM) were similar between MUDT and HIDT, both being higher than for MSDT^13^.

Most published studies on MDS have compared the results of MSDT and MUDT^4^,^6^,^7^,^8^,^9^. Recently, Wang *et al*. used data from the multicenter observational database of the Chinese Bone Marrow Transplant Registry to compare the outcomes between MSDT (n = 226) and HIDT (n = 228). They found that matched sibling donors (MSDs) remain the best donor source for patients with MDS, and that haplo-identical donors (HIDs) can be a valid alternative when an MSD is not available^5^. Until now, there have been few direct comparisons of the results from MSDT and non-MSDT (MUDT and HIDT). For this purpose, in the present study we retrospectively analyzed the data from our center and compared the clinical outcomes between MSDT and non-MSDT with the aim of identifying the prognostic factors that impact OS and OS post-transplant (OSPT). We further analyzed the effect of ATG on the survival of patients with MDS after allo-HSCT.

## Results

### Transplant-related complications

#### Graft-versus-host disease

Acute GVHD (aGVHD) grades I–II had occurred in 28 patients (35.9%) and grades III–IV in 15 (19.2%) at day 100 after transplantation. Ten of these patients had isolated skin aGVHD and 13 isolated intestinal GVHD. Hepatic GVHD occurred in two patients with high bilirubin and transaminase concentrations and aGVHD involved other organs in three patients. The most common type of aGVHD was involvement of two or more organs, such as skin and liver or skin and intestine. Thirty-five of the 67 patients who survived for at least 100 days post-transplantation and were thus evaluable for chronic GVHD (cGVHD).

#### Infectious complications

Bacterial or fungal infections had occurred by the last follow date in 52 of the 78 patients (69.4%) in whom infectious complications were evaluated. The most common type of infection was pneumonia (34 cases), which occurred repeatedly in some cases. Other infectious complications involved the digestive system (presenting with mucositis and diarrhea; 5 cases), sepsis (3 cases), skin and soft tissue (2 cases), central nervous system (1 case), or were complex infections involving two or more organs (7 cases). Cytomegalovirus (CMV) or Epstein-Barr virus (EBV) viremia was detected in 37 patients (21 with CMV, 9 with EBV, and 7 with both CMV and EBV viremia), and haemorrhagic cystitis with BK virus positivity occurred in 20 patients (25.6%).

#### Relapse and NRM

Relapses occurred in 16 patients (18.8%), the cumulative incidence of relapses at the first, second, and third year being 14%, 23%, and 27%, respectively. As shown in Table 1, patients with more than 5% bone marrow blasts at diagnosis and with intermediate karyotype had higher relapse rates after transplantation. In addition, disease that had progressed to advanced stage or evolved to AML was associated with higher risk of relapse after transplantation. However, the incidence of relapse did not differ significantly between patients with MSDT and non-MSDT (18.4% vs. 19.1%, respectively; P = 0.932). Similarly, whether or not ATG was included in the conditioning regimen for GVHD prophylaxis did not significantly impact the risk of disease relapse after transplant. Of note, intensive chemotherapy and/or decitabine therapy did not confer an advantage over supportive care pre-HSCT in terms of risk of relapse after transplantation (25.6% vs. 11.9%, respectively; P = 0.107).

Overall, 38 patients had died, 30 of those deaths being from NRM. The cumulative incidence of NRM at the first, second, and third year were 13%, 23%, and 28%, respectively. NRM did not differ significantly between patients who received MAC conditioning regimens and those who received RIC regimens (P = 0.318). It is noteworthy that patients who received MSDT had higher NRM than those who received non-MSDT. The cumulative incidence of NRM at 1, 2, and 3years was 14%, 37%, and 48% in the MSDT group, which is higher than the 15%, 22%, and 26% in the non-MSDT group. Similarly, NRM occurred significantly less frequently in the ATG (9/39) than in the non-ATG cohort (21/46; P = 0.03).

### Prognostic analysis

**Figure 1:**
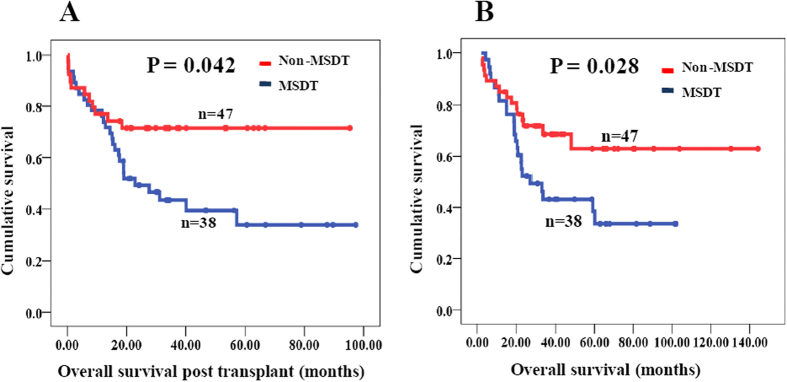
Overall survival post transplant (OSPT) and overall survival (OS) of MDS patients with non-MSDT and MSDT. **(A)** OSPT of MDS patients with non-MSDT and MSDT; (**B**) OS of MDS patients with non-MSDT and MSDT. MSDT: matched sibling donor transplant; non-MSDT: non-matched sibling donor transplant, including matched unrelated donor transplants (MUDT) and haplo-identical donor transplants (HIDT).

For all 85 patients who received allo-HSCT, the median OS was 60.2 months and the probabilities of OS at the first, second, and fifth year 63%, 57%, and 48%, respectively. The median OSPT was 57.2 months and the probabilities of OSPT at the first, second, and fifth year 58%, 55%, and 48%, respectively. To evaluate the impact of donor sources on the OSPT, patients were divided with two groups: MSDT and non-MSDT. The 5-year OSPT rates were 67 and 32% for non-MSDT and MSDT, respectively. The non-MSDT group had a superior survival than the MSDT group, with median OSPT of 84.0 months and 23.6 months, respectively (P = 0.042, Fig. 1A). The results were similar for OS of patients in the non-MSDT or MSDT groups (P = 0.028, Fig. 1B). The incidences of transplant-related complications in the MSDT and non-MSDT groups are shown in Table 2.

The results of univariate analysis of the prognostic factors affecting OS and OSPT are summarized in Table 3. Non-MSDT, peripheral blood stem cells (PBSCs), and use of ATG for GVHD prevention were found to be associated with superior OS and OSPT. Additionally, EBV viremia was associated with both better OS and OSPT according to multivariate analysis. Older age and RIC regimen were predictors of worse OS. However, OS and OSPT were not influenced by sex, WHO classification, IPSS risk, or karyotype at diagnosis. Additionally, differences in pre-HSCT therapies and disease evolution did not significantly impact OS or OSPT.

Multivariate analysis indicated that EBV viremia after transplantation was the only independent factor associated with better OS and OSPT and age was the only independent predictor of shorter OS (Table 4).

### The impact of ATG on survival and transplant related complications

**Figure 2:**
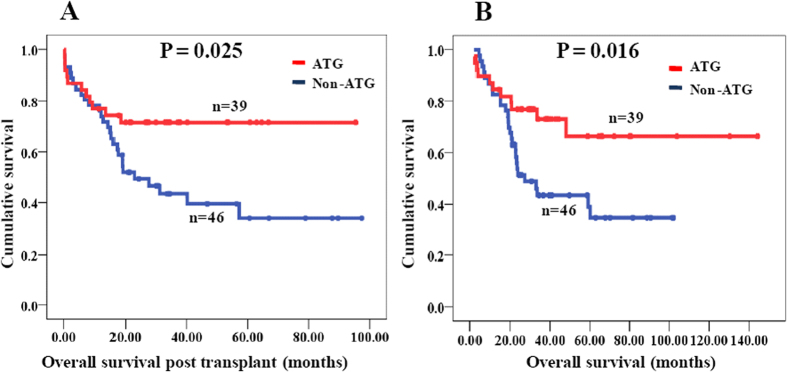
Overall survival post transplant (OSPT) and overall survival (OS) of MDS patients with ATG and non-ATG groups. (**A**) OSPT of MDS patients with ATG and non-ATG groups; (**B**) OS of MDS patients with ATG and non-ATG groups.

**Figure 3:**
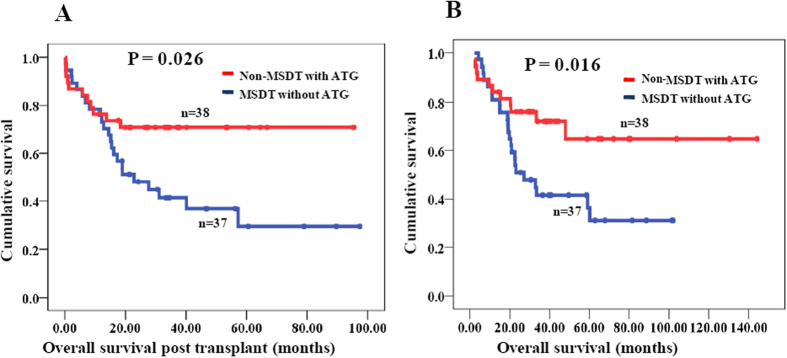
Overall survival post transplant (OSPT) and overall survival (OS) of MDS patients in non-MSDT with ATG group and MSDT without ATG group. (**A**) OSPT of MDS patients in non-MSDT with ATG group and MSDT without ATG group; (**B**) OS of MDS patients in non-MSDT with ATG group and MSDT without ATG group.

According to multivariate analysis, ATG tended to be associated with OSPT and OS (P = 0.075 and P = 0.067); however, this association was not statistically significant. The impact of ATG on OSPT and OS is shown in Fig. 2. Because ATG was usually used for GVHD prevention in the non-MSDT and rarely in the MSDT group, the OSPT or OS of patients who received non-MSDT with ATG (n = 38) and MSDT without ATG (n = 37) were further compared. And the result revealed that using ATG in the conditioning regimen significantly improved both OSPT and OS of in the non-MSDT group (Fig. 3).

Correlations between ATG and other transplant-related complications are shown in Table 5. There was no significant difference in incidence of aGVHD (47.4% vs.62.5%; P = 0.18) or cGVHD (48.5% vs. 55.9%; P = 0.54) between the ATG and non-ATG groups. Similarly, there was no significant difference in relapse rate between these groups. However, patients in the ATG group had a significantly greater rate of CMV or EBV viremia than those in the non-ATG group (Table 5). There was also a non-significant trend to more frequent infection (bacterial and fungal) and haemorrhagic cystitis in the ATG than thenon-ATG patients (Table 5).

## Discussion

Allo-HSCT remains a curative approach to treatment for patients with MDS. In the past few years, significant advances involving all aspects of transplantation have been achieved and clinical outcomes after both MSDT and MUDT have consequently improved^7^. The promising outcomes achieved with HIDT have made it a valid alternative treatment option for patients lacking HLA-matched related or unrelated donors^5^,^17^,^18^. Thus far, few studies have directly compared the clinical outcomes of MSDT and non-MSDT in patients with MDS. In the present study, we found no significant differences in relapse or infection rates between the MSDT and non-MSDT groups. Similarly, these two groups had comparable cGVHD rates. Patients who received non-MSDT had longer OS and OSPT than those who received MSDT. The results of our study differ from those of previous studies, most of which demonstrated better prognosis for MSDT^5^,^7^,^13^. In our study, the superior OS and OSPT in the non-MSDT group were mainly attributable to the lower rates of NRM and aGVHD. Additionally, there was a greater percentage of patients aged less than 40 years in the non-MSDT group (non-MSDT 59.6% vs. MSDT 39.5%); older age has been suggested as a predictor of increased NRM and is correlated with adverse cytogenetic abnormalities^19^,^20^,^21^. In addition, a similar percentage of patients received MSDT during the years 2007–2011 and 2012–2015 (19 cases, 50% vs. 19 cases, 50%), whereas a greater percentage of patients received non-MSDT from 2012–2015 (68.1%, 32 cases) than from 2007–2011 (31.9%, 15 cases). With improvements in supportive care and management of transplant-related complications, the greater proportion of patients receiving non-MSDTs in 2012–2015 than in 2007–2011 may partly explain their superior OS and OSPT in our study.

Promising outcomes of *in vivo* T-cell depletion with ATG for GVHD prophylaxis and have been reported in previous studies of both MUDT and HIDT^13^,^14^,^22^,^23^. Among them were two prospective randomized trials in which patients undergoing MUDT were assigned to standard GVHD prophylaxis with or without ATG. Their findings suggested that the addition of ATG resulted in decreased acute and chronic GVHD; however, the relapse rate and OS were similar regardless of ATG administration^24^,^25^. Recently, another prospective, multicenter, randomized phase 3 study of ATG as part of a conditioning regimen in patients with acute leukemia undergoing MSDT showed that inclusion of ATG resulted in a significantly lower 2-year rate of cGVHD after MSDT than did without ATG (32.2% vs. 68.7%, respectively; P < 0.001). The survival rates were similar in the two groups^26^. In our study, the incidence of aGVHD and cGVHD were lower in the ATG than the non-ATG group; however, this difference was not significant. In line with other studies, the incidences of both CMV and EBV positivity were significantly higher in the ATG than the non-ATG group^26^,^27^. The transplant-related complication of viral reactivation is thought to be correlated with the delayed immune reconstitution after allo-HSCT. Additionally, administration of ATG may increase the risk of viral infections, which can be exacerbated by the use of ATG^28^,^29^,^30^. Interestingly, EBV positivity was a predictor of better OS or OSPT in our study, which may be attributable to the following. First, all EBV viremia were successfully treated in our study. Second, there was a strong correlation between ATG and EBV positivity and ATG suggested better survival in our study.

The dosage of ATG may also significantly influence the clinical outcome after HSCT. In a retrospective study of 465 patients treated with RIC and ATG, doses of ATG exceeding 10 mg/kg were associated with inferior even-free survival and OS^31^. Another study from Hamadani *et al*. reported improved NRM and infection rate with reduction in the dose of ATG from 7.5 mg/kg to 6 mg/kg in patients undergoing RIC–HSCT (including MSDT and MUDT) without compromising the control of aGVHD^32^. Devillier *et al*. reported that an ATG dose of 5 mg/kg in the setting of RIC was associated with a dramatically lower incidence of both aGVHD and cGVHD than a dose of 2.5 mg/kg, without a significant increase in relapse rate in patients receiving MSDT^33^. A recent randomized clinical study enrolled 224 patients with standard-risk hematological malignancies who underwent unmanipulated HIDT; 112 of them received 6 mg/kg ATG and the other 112 10 mg/kg ATG. These researchers found that 6 mg/kg ATG decreased the risk of EBV reactivation compared with 10 mg/kg ATG (9.6% vs.25.3%, P = 0.001); however, the lower-dose treatment was associated with a higher risk of severe aGVHD (16.1% vs.4.5%, P = 0.005)^34^. In our study, most patients received the MAC regimen and a total dose of 10 mg/kg ATG was used pre-HSCT (2.5 mg/kg/day for 4 days). Overall, the optimal dose of ATG in both MSDT and non-MSDT remains unclear and warrants further studies.

The graft versus leukemia (GVL) effect of non-MSDT was not superior to that of MSDT. There was no significant difference in relapse rates between MSDT and non-MSDT in our study, which is in contrast with reports from some previous studies^5^. Although our data should be interpreted cautiously because of our small patient cohort, our results are similar to those of another two studies^13^,^35^. The lower relapse rate in our study may be attributable to the following. First, half the patients in our study were younger than 40 years and most patients received MAC (78/85) conditioning regimens, in contrast to previous studies, which contained a greater percentage of older patients and in which RIC conditioning regimens were administered^4^,^7^. Second, administration of prophylactic donor lymphocyte infusions, which reportedly provide useful immunotherapy for managing post-transplantation relapses^36^, may have contributed to reducing the relapse rates of several patients with refractory disease in our study. Finally, several patients received low dose HMAs for thrombocytopenia after transplantation, which may have played a role in decreasing relapse rates after transplant^37^.

Our study has several limitations. Because we had no data concerning mutations in some patients, we were unable to evaluate the prognostic effect of mutations such as DNMT3a, TP53, and TET2, which have been shown to be correlated with poor prognosis^38^. Another limitation is that insufficient data on ferritin, lactate dehydrogenase, blood transfusion, and coexistence of other comorbidities limited our ability to incorporate these important variables into the analysis. Importantly, our analysis was retrospective and the cohort was small.

In all, despite its retrospective nature and small number of participants, our results indicate that ATG has a beneficial effect in patients with MDS undergoing non-MSDT, being associated with superior survival and a low rate of NRM. These results provide a framework for the refinement and further development of the use of ATG in allo-HSCT, which may have a significant effect on the probability of a favorable outcome.

## Materials and Methods

### Patient characteristics

Data of 85 consecutive patients with MDS who had received allo-HSCT between December 2007 and April 2014 in the first affiliated hospital of Suchow University were analyzed in our study. Informed consent was obtained from the patients before data collection in accordance with institutional guidelines, and the study was approved by the Committees for the Ethical Review of Research at the first affiliated hospital of Suchow University. All methods were performed in accordance with the relevant guidelines and regulations. Patient clinical characteristics were shown in Supplemental file.

Allo-HSCT was considered for patients with higher-risk MDS. Patients with lower-risk MDS (RCUD, MDS-U) with evidence of progression or sustained profound cytopenia (defined as a neutrophil count <0.5 × 10^9^/L and/or platelet count <20 × 10^9^/L) or severe blood transfusion dependence were also considered as candidates for allo-HSCT.

### Donor Selection and HLA-Typing

Human leukocyte antigen (HLA)-matched sibling donors transplants (MSDT) were the first choice for allo-HSCT. If an MSD was unavailable, a suitably matched unrelated donor (MUD) was used, suitable closeness of HLA-matching being defined as >8/10 matching HLA-A, B, C, DR, and DQ loci. Patients without MSD or MUD or whose clinical condition precluded taking the time required for MUD search were eligible for haploidentical donors transplants (HIDT).

### Transplant procedure and GVHD prophylaxis

Granulocyte-colony stimulating factor (G-CSF) (5 μg/kg per day) was used to mobilize bone marrow and peripheral blood. The target mononuclear cell and CD34+cell counts were ≥6 × 10^8^/kg and ≥2 × 10^6^/kg of recipient weight, respectively. Fresh and un-manipulated bone marrow and peripheral blood stem cells (retrieved on Day 5 after G-CSF) were infused into the recipient on the day of collection.

The majority of patients (n = 78, 91.8%) received MAC regimens, the remaining seven receiving RIC regimens. The details of conditioning regimens in MSDT and non-MSDT were shown in supplemental file.

All patients received GVHD prophylaxis consisting of cyclosporine, mycophenolatemofetil and methotrexate.

### Study endpoints, definitions and statistical analysis

Endpoints were OS and OS post transplant (OSPT). OS was defined from the time of diagnosis until death from any cause or until the last follow-up and OSPT from the date of transplant until death from any cause or until the last follow-up. Non-relapse morbidity (NRM) was defined as death from any cause other than disease progression or relapse. Relapse was defined as morphological recurrence of disease. Correlations between the frequencies of different groups were analyzed using the χ^2^ and Fisher’s exact tests. Distributions of OS and OSPT curves were estimated using the Kaplan–Meier method. OS or OSPT were compared between groups using the log-rank test. Prognostic factors with P < 0.05 by univariate analysis were entered into a Cox proportional hazards model to determine the effects of those factors on survival. Differences between groups were considered statistically significant if P values were less than 0.05 in a two-tailed test. All analyses were performed using an SPSS software package (SPSS, Chicago, IL, USA).

## Additional Information

**How to cite this article**: Wang, H. *et al*. Antithymocyte globulin improves the survival of patients with myelodysplastic syndrome undergoing HLA-matched unrelated donor and haplo-identical donor transplants. *Sci. Rep.* **7**, 43488; doi: 10.1038/srep43488 (2017).

**Publisher's note:** Springer Nature remains neutral with regard to jurisdictional claims in published maps and institutional affiliations.

## Supplementary Material

Supplementary Information

## Figures and Tables

**Table 1:** Correlations between relapse rate and clinical variables.

Variables	Not relapse No. (%)	Relapse No. (%)	χ2	P-value
**BM blast** (n = 85)			3.85	**0.050**
<5%	36 (90.0)	4 (10.0)		
≥5%	33 (73.3)	12 (26.7)		
**WHO classification** (n = 85)			3.31	0.191
RCUD/RCMD/MDS-U	34 (89.5)	4 (10.5)		
RAEB-1	17 (77.3)	5 (22.7)		
RAEB-2	18 (72.0)	7 (28.0)		
**IPSS** (n = 84)			2.73	0.098
Low/Int-1	41 (87.2)	6 (12.8)		
Int-2/High	27 (73.0)	10 (27.0)		
**Karyotype** (n = 84)			11.19	**0.004**
Good	43 (91.5)	4 (8.5)	—	
Int	17 (60.7)	11 (39.3)		
Poor	8 (88.9)	1 (11.1)		
**Secondary MDS** (n = 85)			1.01	0.449
Yes	11 (91.7)	1 (8.3)		
No	58 (79.5)	15 (20.5)		
**Progression pre-HSCT**(n = 85)			8.02	**0.005**
Yes	9 (56.2)	7 (43.8)		
No	60 (87.0)	9 (13.0)		
**Donor type** (n = 85)			0.07	0.932
MSD	31 (81.6)	7 (18.4)		
Non-MSD	38 (80.9)	9 (19.1)		
**Conditioning** (n = 85)			0.47	0.491
MAC	64 (82.1)	14 (17.9)		
RIC	5 (71.4)	2 (28.6)		
**ATG** (n = 85)			0.036	0.849
Yes	32 (82.1)	7 (17.9)	—	
No	37 (80.4)	9 (19.6)		
**Therapies pre-HSCT** (n = 85)			2.60	0.107
DAC ± chemotherapy	32 (74.4)	11 (25.6)		
BSC	37 (88.1)	5 (11.9)		

Secondary MDS: MDS that transformed from chronic aplastic anemia; Progression pre-HSCT: MDS that progressed to advanced stage or AML; MSD: matched sibling donors; Non-MSD: including matched unrelated donors (MUD) and haplo-identical donors (HID); MAC: myeloablative conditioning; RIC: reduced intensive conditioning; ATG: Rabbitantithymocyte globulin; DAC: decitabine; BSC: basic supportive care, including erythropoiesis-stimulating agent and blood transfusion.

**Table 2:** Transplant-related complications in MSDT and non-MSDT groups.

Complications	MSDT No. (%)	Non-MSDT No. (%)	χ2	P-value
**Relapse**			0.007	0.932
Yes	7 (18.4)	9 (19.1)		
No	31 (81.6)	38 (80.9)		
**NRM**			4.387	**0.036**
Yes	18 (47.4)	12 (25.5)		
No	20 (52.6)	35(74.5)		
**aGVHD**			6.152	**0.013**
Yes	23 (71.9)	20 (43.5)		
No	9 (28.1)	26 (56.5)		
**cGVHD**			0.323	0.570
Yes	16 (50)	20 (43.5)		
No	16 (50)	26 (56.5)		
**Infection**			0.001	0.970
Yes	21 (65.4)	30 (65.2)		
No	11 (34.4)	16 (34.8)		
**EBV**			6.77	**0.009**
Yes	2 (6.2)	14 (30.4)		
No	30 (93.8)	32 (69.6)		
**CMV**			4.64	**0.03**
Yes	7 (21.9)	21 (45.7)		
No	25 (78.1)	25 (54.3)		
**HC**			0.012	0.914
Yes	8 (25)	12 (26.1)		
No	24 (75)	34 (73.9)		

MSDT: matched sibling donors transplant; non-MSDT: non-matched sibling donors transplant, including matched unrelated donors transplant (MUDT) and haplo-identical donors transplant (HIDT).NRM: nonrelapse mortality; Infection: bacterial and fungal infection; HC: hemorrhagic cystitis.

**Table 3:** Univariate analysis of prognostic factors impacting OSPT and OS.

Variables	No.	Median-OSPT	P-value	Median-OS	P-value
**Gender** (n = 85)			0.438		0.374
Male	53	NA.		48	
Female	32	57.2		NA.	
**Age** (n = 85)			0.056		**0.047**
<40years	43	NA.		NA.	
≥40years	42	27.6		32.9	
**WBC** (n = 85)			0.774		0.691
<4*10E9/L	72	57.2		60.2	
≥4*10E9/L	13	NA.		NA.	
**HB** (n = 85)			0.545		0.566
<100 g/L	70	57.2		59.1	
≥100 g/L	15	40.1		60.2	
**PLT** (n = 85)			0.701		0.781
<50*10E9/L	54	NA.		NA.	
≥50*10E9/L	31	57.2		60.2	
**BM blast** (n = 85)			0.809		0.954
<5%	40	40.1		60.2	
≥5%	45	57.2		NA.	
**WHO classification**(n = 85)			0.554		0.582
RCUD/RCMD/MDS-U	38	40.1		60.2	
RAEB-1	22	NA.		NA.	
RAEB-2	25	57.2		59.1	
**IPSS** (n = 84)			0.632		0.880
Low/Int-1	47	40.1		60.2	
Int-2/High	37	NA.		59.1	
**Karyotype** (n = 84)			0.918		0.965
Good	47	57.2		NA.	
Int	28	40.1		48.0	
Poor	9	NA.		NA.	
**Conditioning** (n = 85)			0.056		**0.043**
MAC	78	NA.		NA.	
RIC	7	8.17		11.2	
**Stem cell source** (n = 85)			**0.004**		**0.004**
Marrow	17	22.9		23.2	
PBSCs	43	NA.		NA.	
Marrow + PBSCs	24	57.2		59.1	
Cord	1	0.4		4.73	
**Therapies pre-HSCT**(n = 85)			0.843		0.902
DAC ± chemotherapy	43	57.2		60.2	
BSC	42	NA.		NA.	
**CR Pre-HSCT**(n = 85)			0.249		0.290
Yes	13	NA.		NA.	
No	72	40.1		60.2	
**Secondary MDS** (n = 85)			0.525		0.563
Yes	12	16.1		33.4	
No	73	57.2		60.2	
**ATG** (n = 85)			**0.025**		**0.016**
Yes	39	NA.		NA.	
No	46	22.9		27.2	
**Progressionpre-HSCT** (n = 85)			0.506		0.520
Yes	16	19.0		22.7	
No	69	57.2		60.2	
**Donor type** (n = 85)			**0.001**		**0.001**
MSDs	38	22.9		27.2	
MUDs	29	NA.		NA.	
HIDs	18	NA.		NA.	
**Infection** (n = 78)			0.053		0.094
Yes	52	27.6		33.5	
No	26	57.2		NA.	
**aGVHD** (n = 78)			0.795		0.877
Yes	43	57.2		59.1	
No	35	NA.		NA.	
**aGVHD** (n = 78)			0.524		0.631
No	35	NA.		NA.	
I-II	25	NA.		NA.	
III-IV	18	19.0		27.2	
**cGVHD** (n = 67)			0.069		0.075
Yes	35	57.2		59.1	
No	32	NA.		NA.	
**HC** (n = 78)			0.922		0.720
Yes	20	NA.		48.0	
No	58	57.2		59.1	
**EBV** (n = 78)			**0.013**		**0.013**
Yes	16	NA.		NA.	
No	62	27.6		33.4	
**CMV** (n = 78)			0.232		0.212
Yes	28	NA.		NA.	
No	50	31.1		48.0	

OS: overall survival; OSPT: overall survival post transplant.

**Table 4:** Multivariate analysis of prognostic factors impacting OSPT and OS.

Variables	OSPT		OS	
	HR (95%CI)	P-value	HR (95%CI)	P-value
Age ≥ 40y			2.17 (1.05–4.46)	**0.035**
RIC Conditioning			1.08 (0.36–3.21)	0.893
PBSCs	0.76 (0.46–1.25)	0.282	0.70 (0.42–1.15)	0.154
MSDT	1.32 (0.73–2.40)	0.360	1.58 (0.83–3.03)	0.167
ATG	0.42 (0.16−1.09)	0.075	0.37 (0.13–1.07)	0.067
EBV	0.22 (0.05–0.95)	**0.042**	0.19 (0.44–0.85)	**0.029**

OSPT: overall survival post transplant; RIC: reduced intensive conditioning; PBSCs: peripheral blood stem cells; MSDT: matched sibling donor transplant; ATG: Rabbitantithymocyte globulin.

**Table 5:** Transplant-related complications in ATG and non-ATG groups.

Complications	Non-ATG group No. (%)	ATG group No. (%)	χ2	P-value
**Relapse**			0.036	0.85
Yes	9 (19.6)	7 (17.9)		
No	37 (80.4)	32 (82.1)		
**NRM**			4.71	**0.03**
Yes	21 (45.7)	9 (23.1)		
No	25 (54.3)	30 (76.9)		
**aGVHD**			1.80	0.18
Yes	25 (62.5)	18 (47.4)		
No	15 (37.5)	20 (52.6)		
**cGVHD**			0.37	0.54
Yes	19 (55.9)	16 (48.5)		
No	15 (44.1)	17 (51.5)		
**Infection**			1.05	0.31
Yes	25 (60)	27 (71.1)		
No	15 (40)	11 (28.9)		
**EBV**			8.53	**0.003**
Yes	3 (7.5)	13 (34.2)		
No	37 (92.5)	25 (65.8)		
**CMV**			4.24	**0.04**
Yes	10 (25)	18 (47.4)		
No	30 (75)	20 (52.6)		
**HC**			0.43	0.52
Yes	9 (22.5)	11 (28.9)		
No	31 (77.5)	27 (71.1)		
