# Supplementary material for: Antithymocyte globulin improves the survival of patients with myelodysplastic syndrome undergoing HLA-matched unrelated donor and haplo-identical donor transplants
Source: Sci Rep. 2017 Mar 6;7:43488. doi: 10.1038/srep43488 (PMC5337979; doi:10.1038/srep43488)
Supplement: Supplementary Information [file srep43488-s1.pdf]

Supplement file

Title: Antithymocyte globulin improves the survival of patients with myelodysplastic syndrome undergoing HLA-matched unrelated donor and haplo-identical donor transplants

Hong Wang[1,+], Hong Liu[1,+], Jin-Yi Zhou[1,+], Tong-Tong Zhang[1], Song Jin[1], Xiang Zhang[1], Su-Ning Chen[1], Wei-Yang Li[1], Yang Xu[1]; Miao Miao[1,*] & De-Pei Wu [1,*]

1) Patient clinical characteristics

Variables	No.
**Sex** (n=85)	
Male/Female	53/32
**Age** (n=85)	
Median (range)	39 (12-59)
**WHO classification** (n=85)	
RCUD	8
RCMD	28
MDS-U	2
RAEB-1	22
RAEB-2	25
**IPSS** (n=84)	
Low	1
Int-1	46
Int2	27
High	10
**Karyotype** (n=84)	
Good	47

Int	28
Poor	9
**Conditioning** (n=85)	
MAC	78
RIC	7
**Stem cell source** (n=85)	
Marrow	17
PBSCs	43
Marrow+PBSCs	24
Cord	1
**Therapies pre-HSCT**(n=85)	
DAC±chemotherapy	43
BSC	42
**CR Pre-HSCT**(n=85)	
Yes	13
No	72
**Secondary MDS** (n=85)	
Yes	12
No	73
**ATG** (n=85)	
Yes	39
No	46
**Progression pre-HSCT**	
(n=85)	
Yes	16
No	69
**Donor type** (n=85)	
MSDs	38
MUDs	29

HIDs	18

MAC: myeloablative conditioning; RIC: reduced intensive conditioning; DAC: decitabine; BSC: basic supportive care, including erythropoiesis-stimulating agent and blood transfusion; Secondary MDS: MDS that transformed from chronic aplastic anemia; ATG: Rabbitantithymocyte globulin; Progression pre-HSCT: MDS that progressed to advanced stage or AML; MSDs: matched sibling donors;MUDs: matched unrelated donors ; HIDs: haplo-identical donors.

2) Conditioning regimens in allo-HSCT

For MSDT, pre-transplant MAC regimens comprised semustine (250mg/m^2, day −10), cytarabine (2g/m^2/d days −9 to −8), busulfan (3.2 mg/kg/d days −7 to −5), cyclophosphamide (1.8 g/ m^2/d days −4 to −3). For non-MSDT (MUDT and HIDT), patients received a regimen identical to that for MSDT apart from receiving higher doses of cytarabine (4 g/m^2/d days −9 to −8). In addition, patients receiving MUDT also received hydroxycarbamide (80mg/kg, day −10). Rabbit ATG (Thymoglobulin; Imtix Sangstat, Lyon, France) was administered to patients receiving MUDT (10 mg/kg total dose, days −5 to −2) and HIDT (10 mg/kg total dose, days −6 to −3).

The RIC comprised semustine (250 mg/m^2, day −10), fludarabine (30 mg/m^2/day, days −10 to −6), cytarabine (1.5 g/ m^2/d days −10 to −6), andbusulfan (3.2 mg/kg/d days −5 to −3). Additionally, ATG was used inpatients receiving MUDT (6 mg/kg total dose, days −4 to −1).
